# Itolizumab regulates activating and inhibitory signals on effector cells, improving their cytotoxicity against CD318+ tumor cell lines

**DOI:** 10.3389/fimmu.2025.1585597

**Published:** 2025-05-05

**Authors:** Cynthia González Muñoz, Rydell Álvarez Arzola, Adanays Calvo Pérez, Milagro de la Caridad Frometa Campanón, Patricia Hernández Casaña, Aymé Fernández-Calienes Valdés, Patricia Lorenzo-Luaces, Zaima Mazorra Herrera, Tania Crombet Ramos, Mayrel Labrada Mon

**Affiliations:** ^1^ Research Division, Center of Molecular Immunology, Havana, Cuba; ^2^ Cellular Biology Department and Biological Sample Bio-Bank, National Institute of Oncology and Radiobiology, Havana, Cuba

**Keywords:** CD6, CD318, ALCAM(CD166), CD5, NK cells, CD8 lymphocytes, immune checkpoint

## Abstract

**Introduction:**

The CD6‐CD318 axis has emerged as a potential target for immuno‐oncology. Recent work has shown that blocking the CD6‐CD318 interaction with a murine anti‐human CD6 antibody increases lymphocyte cytotoxicity. However, several studies have demonstrated the drawbacks associated with the clinical use of murine antibodies and the variability among anti‐CD6 antibodies. Therefore, evidence that the first‐in‐class humanized anti‐human CD6 antibody itolizumab could be used for cancer immunotherapy may be a breakthrough in developing an antitumor clinical approach.

**Methods:**

Phenotypic and functional characterization of peripheral blood mononuclear cells (PBMCs) from healthy donors after challenge with CD318+ cell lines was performed by flow cytometry. In addition, IFNγ was determined by ELISA in culture supernatants. Immunohistochemical analyses of breast tumor samples were also performed.

**Results and Discussion:**

Here, we provide evidence supporting the rationale for itolizumab in cancer immunotherapy. The blockade of the CD6-CD318 interaction by itolizumab increases the cytotoxic capacity of CD8 T and NK cells over CD318+ tumor lines, reverses the NKG2A/NKG2D ratio, and increases granzyme B and IFNγ production. Itolizumab also regulates immune responses by downregulating CD5 expression and upregulating PD-1 and CTLA-4 inhibitory receptors on lymphocytes, which contribute to reducing exacerbated responses and additively enhancing CD318+ tumor cell cytotoxicity when combined with other immunocheckpoint inhibitors. In addition, we report that CD6‐CD318 interaction inhibits lymphocyte proliferation and survival while downregulating CD6 expression on lymphocytes *in vitro* and in human breast cancer tissue samples, reinforcing the role of the CD6‐CD318 axis as an immune checkpoint and highlighting the potential of itolizumab as an immune checkpoint inhibitor. Taken together, our results provide the first evidence linking the blocking of the CD6-CD318 axis by itolizumab with the potentiation of functional properties of lymphocytes, highlighting itolizumab as a novel promising immunotherapy for CD318+ tumors and supporting the relevance of new combinatorial therapies with checkpoint inhibitors.

## 1 Introduction

Over the past decade, immune checkpoint inhibitors (ICIs) have improved the response and survival in many cancer patients (1). However, induction of immune-related adverse effects (irAEs) by ICIs, limits the number of patients achieving durable responses (2), which has greatly increased the scientific community’s interest in searching for new cancer immunotherapies that could suppress autoimmune phenomena. Currently, few molecular targets are considered involved in both pathologies, with CD6 being one of the most studied recently (3).

CD6 is a membrane glycoprotein expressed by all thymocytes, mature T cells, NK cells, and specific B cell subpopulations (4–6). CD6 comprised three extracellular scavenger receptor cysteine-rich (SRCR) domains, a transmembrane region, and a cytoplasmic tail susceptible to phosphorylation and interaction with effector signaling molecules (7). The molecule has been involved in several physiological processes, such as immune synapse stabilization, sustained T cell proliferation, and leukocyte adhesion and transmigration processes (8–10). Targeting CD6 has also been shown to attenuate T cell responses by molecular mechanisms not yet determined probably involving binding to inhibitory tyrosine phosphatases, suppressing tyrosine kinases signaling activity, or directly interacting with the inhibitory receptors like CD5 (11, 12). The function of CD6 as a signalosome that modulates both activating and inhibitory pathways contributes to the diversification of TCR/CD3 explaining the dual functions reported for the molecule (13, 14).

Several ligands for the CD6 molecule (CD6L) have been reported. Among the best characterized are activated leukocyte cell adhesion molecule (ALCAM or CD166) and CD318 (15, 16), although interactions with galectins 1 and 3 and, more recently, CD44 have also been reported (17, 18). Tumor cells overexpress most of the reported ligands for CD6, and their interaction plays an important role in tumor progression and invasion, as well as in tumoral immuno-evasive mechanism. Thus, the diverse properties of CD6 and its ligands can be exploited in novel immunomodulating potential (19, 20). CD6 targeting is an interesting candidate for alternative or complementary cancer immunotherapies. The soluble form of extracellular domains of CD6 has been successfully used in different mouse cancer models (21). Recently, it has been demonstrated that blockade of CD6-CD318 interactions with a murine anti-human CD6 antibody induced increased cytotoxic capacities of CD8+ T cells and NK cells in *in vitro* and *in vivo* models (22, 23). However, the use of murine antibodies in patients has shown high immunogenicity, hastened clearance, and poor recruitment of human immune effector mechanisms, which are drawbacks for their clinical use (24).

Itolizumab (also known as T1h) is a first in-class humanized nondepleting IgG1 anti‐CD6 monoclonal antibody (Mab) produced by the Centre of Molecular Immunology (CIM) (25). Itolizumab is the only anti-CD6 antibody approved for use in patients and has shown promising clinical-stage results in various immune-mediated diseases with favorable safety and efficacy results in Cuba and India (25). Itolizumab, similar to other anti-CD6 antibodies specifically recognizes CD6 domain 1. However, several studies have reported differences in the binding epitope and variable functional effect of commonly used anti-CD6 antibodies (26, 27). It is remains to be proven whether the use of different anti‐CD6 Mab, despite their specific characteristics, will lead to the same outcome in cancer immunotherapy. This paper shows that the humanized anti‐CD6 antibody itolizumab, similar to murine UMCD6, enhances the cytotoxic capacities of immune cells by blocking the negative signals associated with CD318 expressed in tumor cells. Moreover, we demonstrate that itolizumab regulates immune cell responses, avoiding exacerbated or anergic responses to stimuli, representing an advantage over other ICI therapies. We also show the first evidence of the synergistic effect of target CD6 and PD‐1 in antitumor responses.

## 2 Materials and methods

### 2.1 Cell culture and tumor cell line

The following cell lines were used in functionality experiments: human cancer cell lines MDA-MB-231 (triple-negative breast epithelial adenocarcinoma), MCF-7 (breast epithelial adenocarcinoma), NCI-H460 (large cell lung epithelial carcinoma), SKOV-3 (ovarian epithelial adenocarcinoma) and HCT-116 (colorectal epithelial carcinoma). All cell lines were obtained from American Type Culture Collection (ACTT, USA) and cultured in Modified Dulbecco medium (DMEM) (GIBCO, USA) supplemented with 10% fetal bovine serum (FBS) (GIBCO, USA) Cell lines were maintained at 37°C, in an atmosphere of 5% CO_2_. After reaching near confluency (85%) cells were trypsinized and collected for flow cytometry analysis or the respective assays.

### 2.2 Antibodies

For the *in vitro* experiments, the following MAbs were used: itolizumab, a human IgG1 specific for the domain 1 of the human CD6 molecule (CIM, Cuba); a control human IgG1 isotype antibody (DDXCH01P-100, Novus Biological, USA); a commercial neutralizing antibody specific for human CD318 (3A11, Merck, USA) and a commercial neutralizing antibody specific for human ALCAM (105901, Bio‐Techne, USA) and a humanized anti‐PD‐1 antibody pembrolizumab (Keytruda, USA).

All antibodies used in flow cytometry experiments were purchased from BD Biosciences (San Jose, USA), and BioLegend (San Diego, USA). Anti-human antibodies used for this study consisted of CD45-FITC (clone 2D1), CD3-APCCy7 (clone SK7), CD4-Alexa fluor (clone SK3), CD8-PE (clone SK1), (clone 301040), CD6‐APC (clone BL CD6), CD6-PE (clone MEM-98), CD5-PERCPCy5.5 (clone L12F12), CD69-PECy7 (clone FN50), NKG2D-FITC (clone 1D11), Granzyme B‐FITC (clone GB11), CTLA‐4‐FITC (clone BNI3), NKG2A-PEDazzle594 (clone S19004C), PD-1-PECy7 (clone EH12.2H7), ALCAM-PERCPCy5.5 (clone QA17A16), CD318-PE (clone CUB1). 7-Aminoactinomycin D (7AAD) staining solution (130-111-568, Miltenyi Biotec, USA) was used as a cell death marker.

Human CD6 (clone 5A10A2, MA5‐38488, ThermoFisher, USA) and CD318 (polyclonal, ab1377, Abcam, USA) specific mouse mAbs were used for immunohistochemical (IHC) evaluations. Horseradish peroxidase-conjugated mouse immunoglobulin G (IgG)-specific from Santa Cruz Biotech (sc-2031, Santa Cruz, USA) was used as a secondary antibody.

### 2.3 PBMCs isolation and subpopulation enrichment

For *in vitro* experiments, peripheral blood mononuclear cells (PBMCs) were purified from buffy coats of anonymous healthy donors between 25 and 45 years old, at the blood donation center at the Center for Medical and Surgical Research (CIMEQ) in Havana. Donors were age and sex-matched. The study was performed according to the principles of the Declaration of Helsinki. Donors gave their informed consent and the institutional ethical committee approved the investigation of the human samples. Clinical data are summarized in **Supplementary Table 1**.

PBMCs were isolated using density gradient centrifugation (Ficoll-Paque™ PLUS, GE Healthcare, USA). The PBMCs layer was retrieved, washed two times with phosphate-buffered saline solution (PBS) (GIBCO, USA), counted using a hemocytometer, and resuspended in Roswell Park Memorial Institute medium (RPMI‐1640) (GIBCO, USA) supplemented with 10% SFB, 1mM sodium pyruvate, 50µM mercaptoethanol, and 1mM L-glutamine. Viability was measured by trypan blue dye (GIBCO, USA) exclusion assay.

Isolated PBMCs or particular subpopulations were used for tumor-killing assays. CD8+ and CD4+ T-cells, and NK (CD56+) cells were isolated from the buffy coat by negative selection using commercial purification kits (CD8+ T Cell Isolation Kit human and CD4+ T cell Isolation Kit human, Miltenyi Biotec; EasySep™ Human NK Cell Isolation Kit, STEMCELL Technologies), and following the manufacturer’s instructions.

### 2.4 Tumor cell killing assays

The tumor cell-killing assays used in this work were optimized based on kinetic killing assay and itolizumab dose-response curves (**Supplementary Figure 1**). Flow cytometry analysis of tumor cell death after 120 hours of co‐culture with treated PBMC was selected as the best method to evaluate the effect of itolizumab on tumor cell killing. Briefly, tumor cell lines (2.5 × 10^4^/well) were seeded in U-bottom 96-well plates. PBMCs or specific subpopulations were left untreated or preincubated overnight with itolizumab or IgG1 isotype control. Then, immune cells were added (5 × 10^4^/well) to tumor cell-containing wells and co-cultured for 120 hours, after which all cell types were harvested and washed for flow cytometry staining. To discriminate between effector cells (PBMCs) and target cells (tumor cell lines), extracellular labeling with fluorophore-conjugated anti-CD45 antibodies (30 minutes at 4 ˚C) was performed. After a second washing step, cells were incubated with 7AAD for 10 minutes in the dark just before reading on a flow cytometer (Gallios™, Beckman Coulter).

Tumor cell-killing assay was also used to evaluate whether the effect of itolizumab was determined by blocking CD6-CD318 or CD6-ALCAM interactions. Briefly, cell lines were pre-incubated with 10 µg/ml of neutralizing anti-ALCAM, neutralizing anti-CD318, or IgG1 isotype control and challenged with untreated PBMCs. PBMCs were pre-incubated with 10 µg/ml itolizumab or IgG1 isotype control and challenged with untreated MDA-MB-231 cell line. Tumor cell line lysis was assessed by flow cytometry after 120 hours of incubation as described above.

### 2.5 ICI and itolizumab combined therapy

To evaluate the effect of combinational therapies targeting CD6 and PD-L1, the cytotoxic effect induced by itolizumab, pembrolizumab, and the combination of both in PBMCs challenged with the MDA-MB-231 breast tumor cell line was assessed. For this purpose, PBMCs were pre-incubated with 10 μg/ml isotype control, itolizumab, pembrolizumab, or the combination of these controls and challenged with the MDA-MB-231 breast tumor line using the same protocol described for tumor-killing assay.

### 2.6 T-cell proliferation assay

For *in vitro* proliferation assay, PBMCs from healthy individuals were labeled with 1 mM CFSE (Invitrogen™ CellTrace™ CFSE Cell Proliferation Kit, USA), followed by incubation with anti-CD3/CD28/CD2-beads (bead: cell ratio of 2:1, Miltenyi Biotec) in 96-well plates previously coated with 10 µg/ml of recombinant human CD318 (rhCD318, 112311, Abcam) or recombinant human ALCAM (rhALCAM, 768804, Biolegend). Bead unstimulated cells and PBS-coated wells were used as controls. PBMCs were incubated for 96 hours at 37 °C, 5 % CO_2_, and T cell proliferation was assessed by dilution of CFSE using flow cytometry.

### 2.7 Transwell indirect co-culture assay

A modified Boyden chamber assay was performed to evaluate the effect of secretory factors and soluble molecules from tumor cells over immune cells. Briefly, MDA‐MB‐231 (2 x10^4^ cells/cm^2^) cells were seeded in Matrigel-coated transwell inserts (24 well-clear, 0.4 μm pore sized PET membrane, Corning, USA). PBMC (2 x10^5^ cells/mL) were then plated in the corresponding bottom 24 well-plates. After 120 hours of indirect co-culture PBMC were collected for flow cytometry analysis. PBMC cultured alone and direct co-culture of MDA-MB-231 cells and PBMC were used as control.

### 2.8 Flow cytometry

Phenotypic and functional characterization of PBMCs, purified CD8+ T, and NK cells after challenge with CD318+ cell lines was performed by flow cytometry. Briefly, co-cultured cells were incubated with the fluorescent antibodies listed above, using CD45 to distinguish effector cells from target cells. To assess the production of granzyme B, the cells were permeabilized with the FOXP3/transcription factor staining buffer kit (ThermoFisher Scientific, USA) according to the manufacturer’s instructions.

For all flow cytometry experiments performed, the MAbs used were pre-titrated and the incubation period for labeling the cells was always 30 min at 4˚C. Blocking of unspecific binding of antibodies to the human Fc receptor-expressing cells was achieved using an FcR blocking reagent (Miltenyi Biotec).

Samples were acquired on Beckman Coulter Gallios™ flow cytometer.

### 2.9 Immunoadsorbant assay

The levels of soluble human IFNγ in the supernatant of co-cultures between purified CD8+ T or NK cells and tumor cell lines were quantified with the human IFN-gamma Quantikine ELISA Kit (R&D Systems, USA), respectively, following the manufacturer’s instructions.

### 2.10 Immunohistochemistry

Formalin-fixed and paraffin-embedded (FFPE) breast tumor samples from biopsies at the diagnosis of 117 patients were obtained from the pathology department of the National Institute of Oncology and Radiobiology of Havana, Cuba. The study was performed according to the principles of the Declaration of Helsinki. Patients gave their informed consent and the institutional ethical committee approved the investigation of the human samples. Clinical data are summarized in **Supplementary Table 1**.

For immunohistochemistry (IHC), hematoxylin and eosin (H&E) staining sections of 4 μm were used. Once dried, the sections were treated with OTTIX plus solution and OTTIX shaper solution (Diapath) to dewax and rehydrate the sections. Antigen retrieval was performed using pH 6 solutions at 98°C for 20 min. Next, the endogenous peroxidases and non-specific-binding sites were blocked using 3% H_2_O_2_ and Protein‐Block solution (DAKO Agilent technologies) respectively, for 10 min. Sections were stained for anti-CD6 (1:200) and anti-CD318 (1:200).

### 2.11 Data analysis

IHC analyses were performed using the Imagescope software. The staining intensity was scored as 0–3: 0 (negative), 1 (low), 2 (intermediate) and 3 (high).

For cytometry data analyses, all samples were characterized according to fluorescence intensity (MFI) and forward and side scattering. The gating strategy excluded doublets (using classical gating strategy) and dead cells using 7AAD. All data obtained were analyzed using FlowJo v10.8.1 (Tree Star Inc., Oregon, USA).

GraphPad Prism 7 (GrahPad Software Inc., California, USA) was used for statistical analysis. Normality and homogeneity of variance were tested for all samples using the Shapiro-Wilk and Bartlett tests, respectively. Statistical comparisons were performed using the parametric unpair Student’s t-test (two-tailed) for comparisons between two groups or the combination of an un pair one-way ANOVA with Tukey’s test or Kruskal-Wallis test with Dunn’s multiple comparisons test for comparisons between more than two groups. Unless otherwise indicated, data are shown as median with 95% confidence interval. Differences were considered significant when *p<0.05, **p<0.01, ***p<0.001 and ****p<0.0001.

## 3 Results

### 3.1 Itolizumab enhanced tumor cell killing by PBMCs challenged with human tumor cell lines

In 2021, Ruth et al. reported that UMCD6, a mouse antibody specific for domain 1 (D1) of human CD6, increased the cytotoxic capacity of PBMCs against tumor cell lines (22). Differences in recognition epitopes, affinity, and functions between UMCD6 and itolizumab have been reported (26). Therefore, this work aimed to determine if itolizumab, a first-in-class humanized antibody that recognizes a different epitope of CD6D1, showed a similar capacity described for UMCD6.

Since it is established that CD6 ligands ALCAM and CD318 levels are increased in malignant tumors (19), expression levels of CD6, CD318, and ALCAM were assessed on five human tumor cell lines: MDA-MB-231, MCF-7, NCI-H460, HCT-116, and SKOV-3. None of the tumor cells tested were CD6 positively stained. However, the expression of its ligands CD318 or ALCAM was observed. MDA-MB-231, NCI-H460, SKOV-3, and HCT-116 cell lines showed high levels of CD318 expression, while only MCF-7 was CD318 negative. Regarding ALCAM, all tumor cells were positive for the molecule with variable expression levels (**Supplementary Figure 2A**). CD6 expression levels were also measured in immune cells of healthy donors. In agreement with what is described for the molecule (4), CD6 was highly expressed on both CD4+ T and CD8+ T lymphocytes. NK cells were also CD6+, although to a lesser extent than T lymphocytes (**Supplementary Figure 2B**).

**Figure 1:**
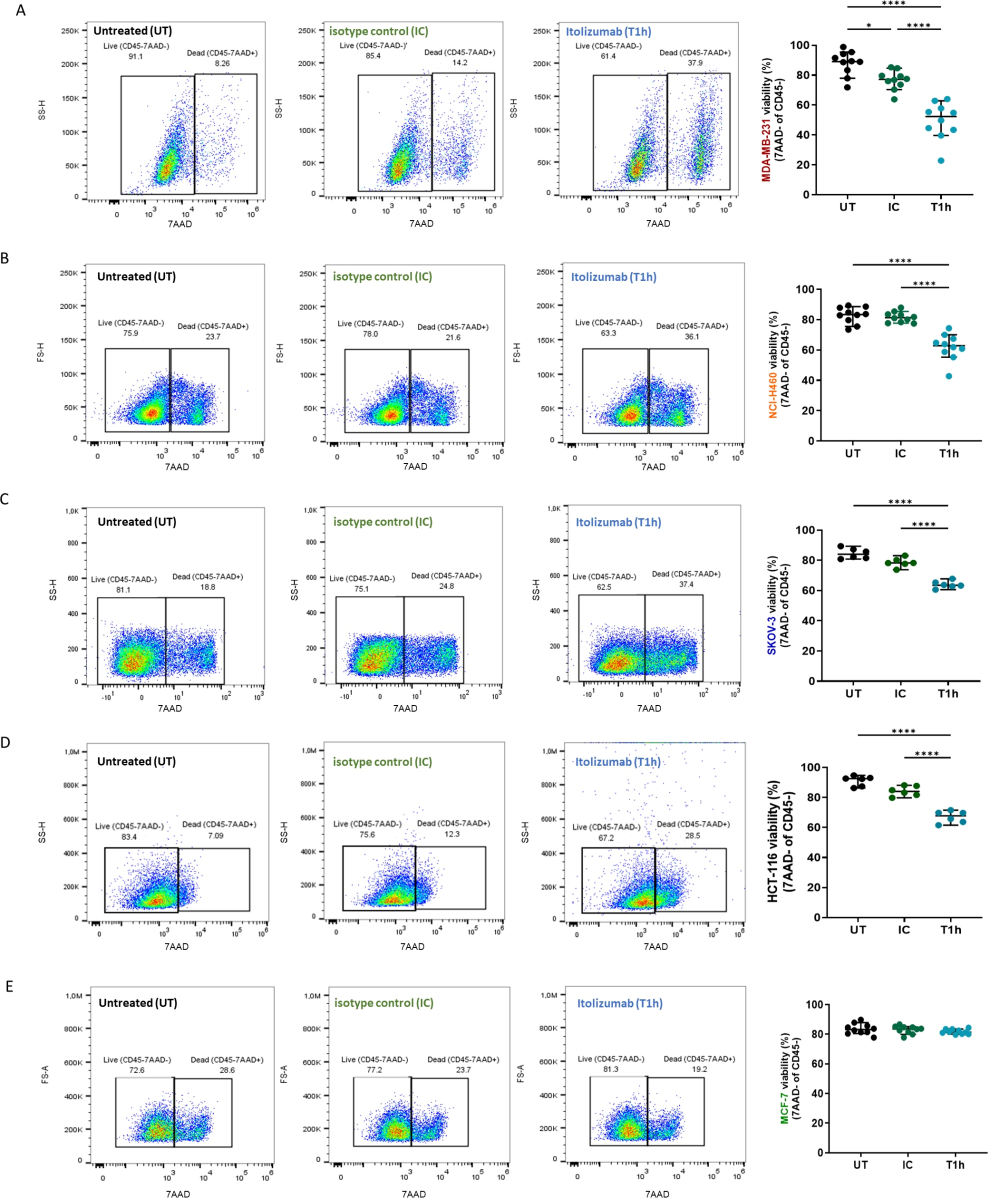
Itolizumab enhanced tumor cell killing by PBMC challenged with CD318+ tumor cell lines. PBMC were maintained untreated (UT, black dots) or were pre-incubated with 10 µg/mL of isotype control (IC, green dots) or itolizumab (T1h, blue dots) and challenged with **(A)** MDA-MB-231 (n=10), **(B)** NCI-H460 (n=10), **(C)** SKOV-3 (n=6), **(D)** HCT-116 (n=6) and **(E)** MCF-7 (n=10) human tumor cell lines. Tumor cell lysis was measured using 7AAD staining by flow cytometry. Representative dot plots for each condition and percentage of tumor cell viability in the co-cultures for each donor are shown. Data are depicted as median ± 95% confidence interval. Statistical analysis was performed using one-way ANOVA and Tukey’s multiple comparisons test, or Kruskal-Wallis test and Dunn’s multiple comparisons test, both for unpaired data. Only statistical significance is shown in the graphs, with *p ≤ 0.05, and ****p ≤ 0.0001.

PBMCs isolated from blood samples of healthy donors were pre-incubated with an IgG1 isotype control or itolizumab and challenged with MDA-MB-231, NCI-H460, HCT-116, SKOV-3, and MCF-7 cancer cell lines. The results showed that itolizumab significantly enhanced cancer cell killing by PBMCs challenged with CD318+ tumor cells (**Figure 1A–D**). However, this effect was undetected for CD318- tumor cell line MCF-7 (**Figure 1E**). The increase of the PBMC killing capacity varied within the cell lines and was positively correlated with CD318 expression and inversely correlated with ALCAM expression (**Supplementary Figure 2C**).

### 3.2 Blockade of CD6-CD318 interaction and not CD6-ALCAM boosted PBMCs-mediated tumor cell killing

**Figure 2:**
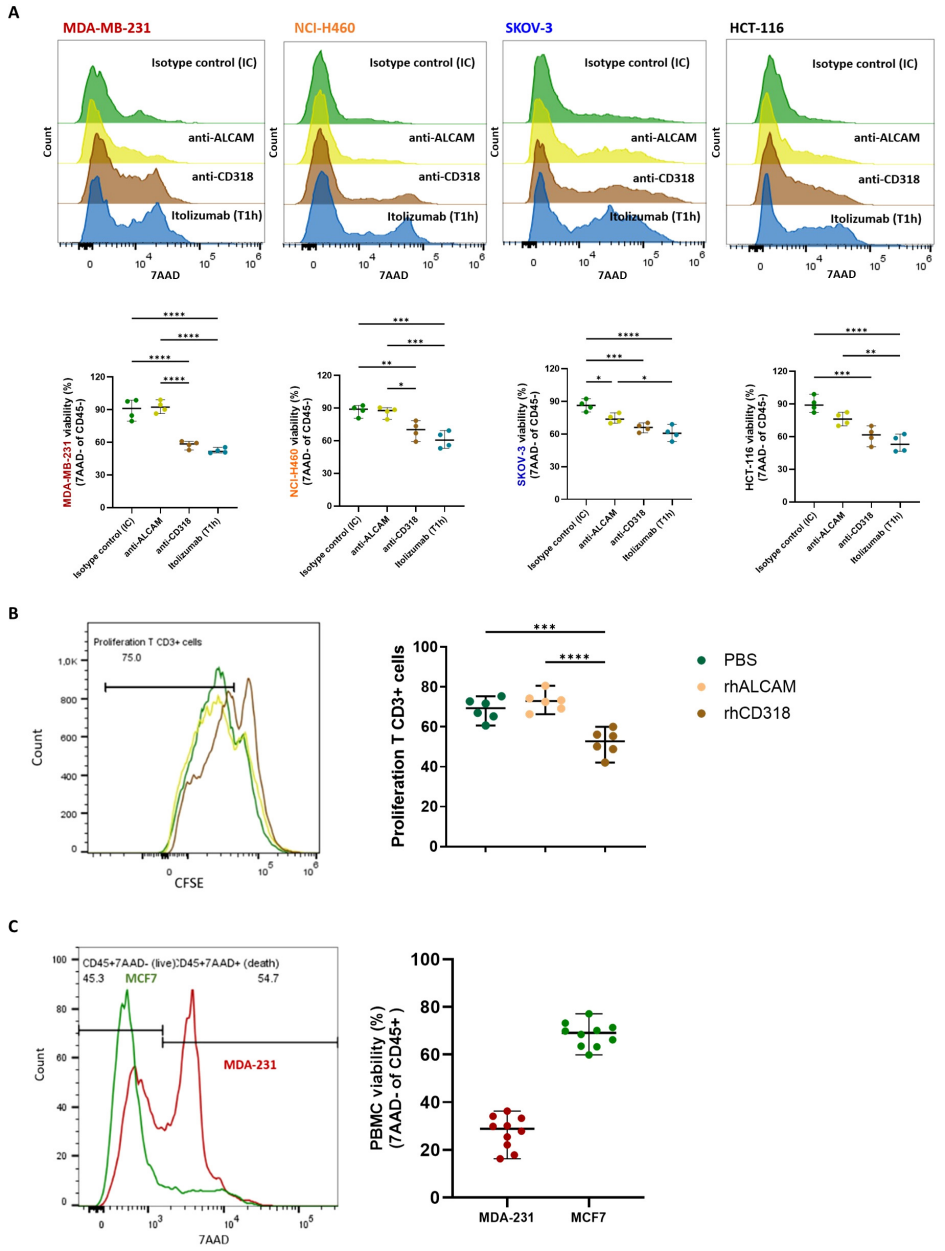
Itolizumab increased the tumor cell killing capacity of PBMC by blocking the inhibitory effects associated with CD6-CD318 interaction. **(A)** PBMC and MDA-MB-231, NCI-H460, SKOV-3 and HCT-116 cell lines (n=4) were pre-incubated with 10 µg/mL of isotype control (IC, green), itolizumab (T1h, blue), or neutralizing antibodies specific for CD318 (brown) or ALCAM (yellow). Effector and target cells were then co-cultured, and tumor cell lysis was measured using 7AAD staining by flow cytometry. **(B)** CFSE-labeled T-cells (n=6) were activated with antiCD3/CD28 beads and incubated with 10ug/mL of pre-coated human recombinant CD318 (brown), ALCAM (yellow), or PBS (green) as control. **(C)** Immune cell viability was measured on isotype control treated-PBMC co-cultured with human tumor cell lines MDA-MB-231 (n=10, red) and MCF-7 (n=10, green) using flow cytometry. Representative histograms or dot plots for each condition, and individual viability percentage and CFSE dilution are displayed. Data are depicted as median ± 95% confidence interval. Statistical analysis was performed using the Kruskal-Wallis test and Dunn’s multiple comparisons test with unpaired data. Only statistical significance is shown in the graphs, with *p ≤ 0.05, **p ≤ 0.01, ***p ≤ 0.001, and ****p ≤ 0.0001.

Considering that the tumor cell lines do not express CD6, which excludes antibody-mediated cytotoxicity such as antibody-dependent cellular cytotoxicity (ADCC), and that itolizumab-mediated killing was only observed in those tumors expressing CD318, it was hypothesized that the increased cytotoxic capacity of PBMCs in co-cultures, in the presence of itolizumab, depends on the blockade of the interaction of CD6 with this ligands. Accordingly, whether the blockade of CD6-ALCAM or CD6-CD318 interaction influenced the cytotoxic mechanism triggered by itolizumab was investigated. Blocking CD6-CD318 interaction with itolizumab or anti-CD318 antibody showed similar augmented tumor cell killing by PBMCs challenged with CD318+ cells. However, blocking CD6-ALCAM interaction with a neutralizing anti-ALCAM antibody did not significantly affect the viability of MDA-MB-231, NCI-H460 and HCT-116 tumor cell lines, and it only slightly decreased the viability of SKOV-3 (**Figure 2A**), suggesting that the enhanced effect on the killing activity of the PBMCs results from the blockade of CD6-CD318 interaction.

### 3.3 CD6-CD318 interaction yields inhibitory signals in immune cells

Since blocking CD6-CD318 interaction by itolizumab triggers cytotoxic responses of effector cells, it is plausible that the expression of CD318 in cancer cells induces inhibitory signals over the immune cells, acting as a tumor immunosuppressive mechanism. To assess this hypothesis, the role of soluble rhCD318 and rhALCAM on T‐cell proliferation was evaluated by a CFSE proliferation assay. Activated T-cell populations displayed reduced proliferative capacities when exposed to rhCD318, compared with rhALCAM and PBS (**Figure 2B**). This result suggests the inhibitory role of CD318 in T-cell proliferative responses.

The impact of the CD6-CD318 interaction on PBMC viability was also evaluated. Compared to MCF-7, PBMCs challenged with CD318+ cell lines had reduced viability (**Figure 2C**), indicating that CD318 but not ALCAM may also impair immune cell viability.

**Figure 3:**
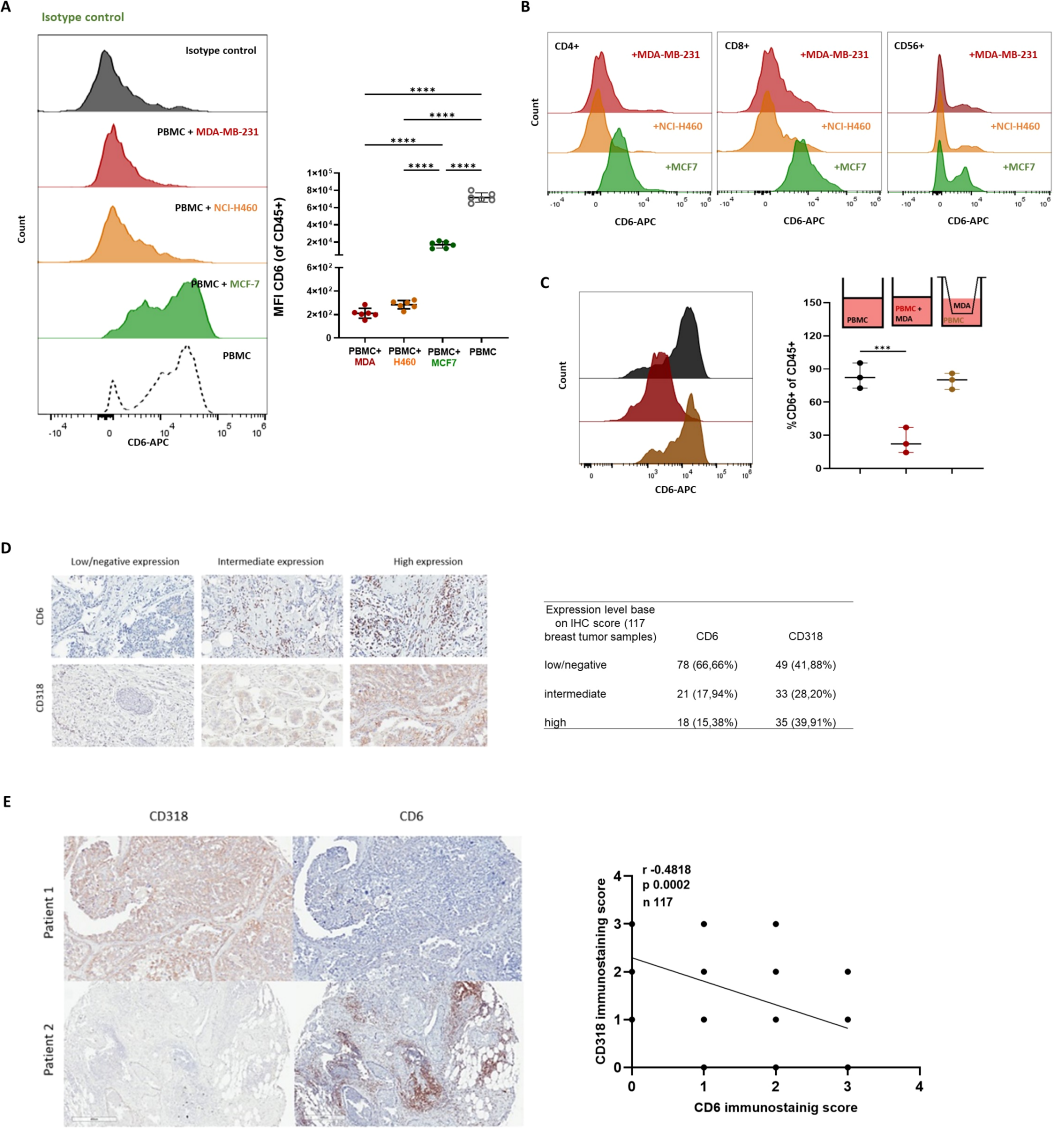
CD6-CD318 interaction induced CD6 downmodulation. Isotype control treated PBMC were co-cultured with human tumor cell lines MDA-MB-231 (red), NCI-H460 (orange), and MCF-7 (green). CD6 loss was measured on PBMC (n=6) using flow cytometry. **(A)** Representative histograms of CD6 expression and individual values of CD6 MFI on isotype control treated PBMC challenged with tumor cell lines. **(B)** Representative histograms of CD6 expression on isotype control treated CD4+ and CD8+ T-cells and NK (CD56+) cells challenged with tumor cell lines. **(C)** Representative histograms and frequencies of CD6+ cells on isotype control treated PBMC challenged with tumor cell lines and co-cultured in a modified Boyden chamber with a transwell membrane of 0.4μm. Data are represented as median ± 95% confidence interval. Statistical analysis was performed using one-way ANOVA with unpaired data and Tuckey’s multiple comparison test. Only statistical significance is shown in the graphs, with ***p ≤ 0.001, and ****p ≤ 0.0001. (**D).** Representative IHC staining with CD318 and CD6 in breast tumor tissue samples (n 117) and relation of patients with low/negative, intermediate, and high staining scores for both molecules. (**E).** Pearson’s correlation analysis of CD318 expression and CD6+ infiltrated on breast tumor tissue samples by IHC.

Moreover, since CD6-dependent signals are involved in several immune responses including proliferation, activation, and survival, CD318 may induce its inhibitory functions by downmodulating CD6 expression on immune cells. CD6 expression was measured on PBMCs treated with isotype control and challenged with CD318+ (MDA-MB-231 and NCI-H460) and CD318- (MCF-7) tumor cell lines. Higher loss of CD6 expression was observed in co-cultures with CD318+ tumor cell lines, compared with MCF-7 in the presence of isotype control (**Figure 3A**) pointing to a CD6 loss mechanism induced by CD6-CD318 association. Downmodulation of CD6 in co-cultures with CD318+ tumor cell lines was also observed in CD4 and CD8+ T cells, and NK cells (CD56+) (**Figure 3B**).

To evaluate if the reduction of CD6 expression on PBMC is due to direct cell-cell contact or if it is caused by soluble forms of CD318, PBMC and tumor cells were co-cultured separately using a modified Boyden chamber with a transwell membrane of 0.4μm pore that allowed migration of soluble factors but not cells. CD6 expression was not altered on immune cells (CD45+) co-cultured with MDA-MB-231 lines in the transwell system (**Figure 3C**), suggesting that CD6 downmodulation is caused by the direct interaction of PBMC and tumor lines and not by the presence of soluble forms of CD318.

Interestingly, although itolizumab-treated PBMCs co-cultured with tumor cell lines show similar CD6 downmodulation **(Supplementary Figure 3A**), the functional responses are different in each condition, suggesting that CD318 and itolizumab decrease CD6 expression by a different mechanism that could trigger alternative outcomes. CD6 loss due to itolizumab receptor occupancy was excluded by dissociating the antigen-antibody interaction with a glycine‐HCL buffer pH 2.5 (**Supplementary Figure 3B).**

To study whether CD6 loss was also detected *in vivo*, CD318 expression patterns and CD6+ lymphocytic infiltrate on tumors were evaluated by IHC using tissue microarrays (TMA) containing breast tumor samples from 117 patients. Tissue samples showed variable intensity of CD318 expression and levels of CD6+ infiltrate that also showed variable signals (**Figure 3D**).

The analysis of the samples determined that in 59.80% of the cases, there was an inverse correlation (r -0.4818) between the expression levels of CD318 and the CD6 positive immune infiltrate, with high CD318+/low CD6+ signaling predominantly over low CD318+/high CD6+ staining (39.21% vs 20.58%, **Figure 3E**). Higher co-expression levels of CD318 and CD6 were only observed in 16.66% of the cases, while in the remaining 28.43% of samples, positive staining was weak or negative for both molecules.

### 3.4 Itolizumab augmented activation of CD8+ T and NK cells challenged with tumor cell lines

**Figure 4:**
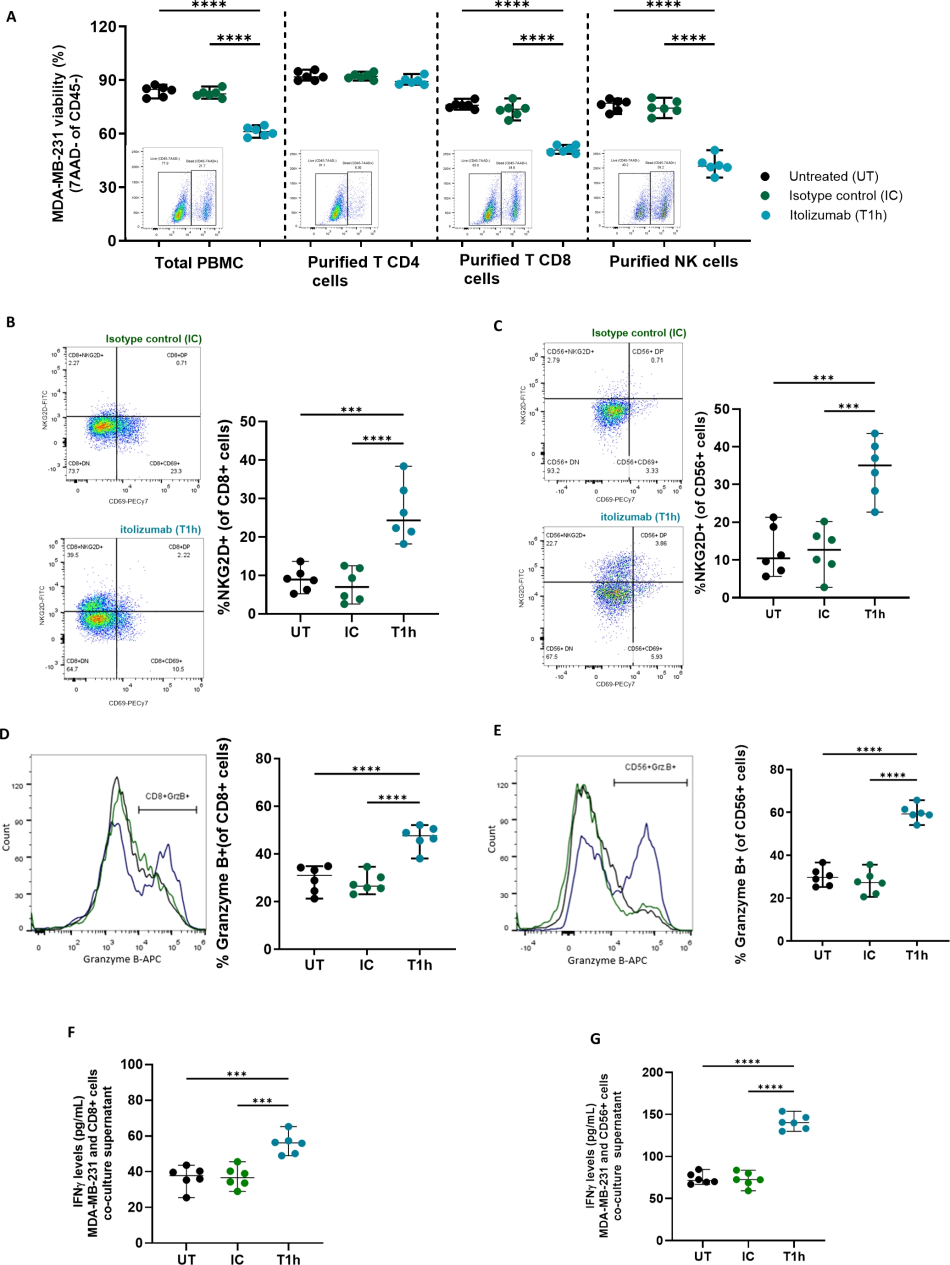
Itolizumab enhanced tumor cell killing by activating CD8+ and NK cells challenged with CD318+ tumor cell lines. PBMC and purified CD4+ and CD8+ T-cells and NK cells were maintained untreated (UT, black) or were pre-incubated with 10 µg/mL of isotype control (IC, green) or itolizumab (T1h, blue) and challenged with MDA-MB-231 (n=6). **(A)** Tumor cell viability was measured using 7AAD staining by flow cytometry. The frequency of NKG2D+, CD69+, and granzyme B+ in **(B, D**) CD8+ T and **(C, E**) NK cells in the co-cultures were determined by flow cytometry. Representative dot plots or histograms of each condition and individual percentage of positive cells are displayed. IFNγ levels on co-culture supernatants of MDA-MB-231 with enriched CD8+ T **(F)** and NK cells **(G)** were quantified by ELISA. Individual values of IFNγ concentration (pg/mL) per donor are shown. Data are depicted as median ± 95% confidence interval. Statistical analysis was performed using one-way ANOVA and Tukey’s multiple comparisons test. Only statistical significance is shown in the graphs, with ***p ≤ 0.001, and ****p ≤ 0.0001.

Since itolizumab boosts the cytotoxic capabilities of immune cells, it was evaluated which subpopulations within PBMCs were involved in the antitumoral effect of itolizumab. To compare the results with those reported for UMCD6, the MDA-MB-231 cell line was chosen. Isolated NK cells and CD8+ T lymphocytes treated with itolizumab and co-cultured with MDA-MB-231 tumor cells showed higher cytotoxic capacities than total PBMCs. On the other hand, isolated itolizumab-treated CD4+ T cells did not increase cancer cell lysis (**Figure 4A**).

To evaluate if itolizumab triggers a direct activating mechanism in cytotoxic cells, activation markers’ expression and cytokine production on isolated NK and CD8+ T lymphocytes challenged with CD318+ cell lines were measured. The data revealed that itolizumab increased the frequency of NKG2D+ within CD8+ T cells (**Figure 4B**) and NK cells (**Figure 4C**) challenged with MDA-MB-231 cell line compared with isotype control. Likewise, itolizumab also augmented granzyme B and IFNγ production by CD8+ T and NK cells (**Figures 4D–G**) in this *in vitro* model. CD8+ T and NK cells co-cultured with the other CD318+ cancer cell lines NCI-H460, HCT-116, and SKOV-3 displayed similar outcomes (**Supplementary Figure 4**).

Itolizumab-treated lymphocytes in mono-cultures (**Supplementary Figure 5A**) and co-cultures with MCF-7 (**Supplementary Figure 5B**) also showed increased activation levels compared to isotype control. This result suggests that although the modulation of CD6 with itolizumab activates cytotoxic lymphocytes, the enhancement of tumor cell killing with the antibody is dependent on the disruption of the CD318 inhibitory signal on CD8+ T cells and NK cells by blocking the CD6-CD318 interaction.

### 3.5 Itolizumab regulates the levels of inhibitory receptors on immune cells challenged with tumor cell lines

**Figure 5:**
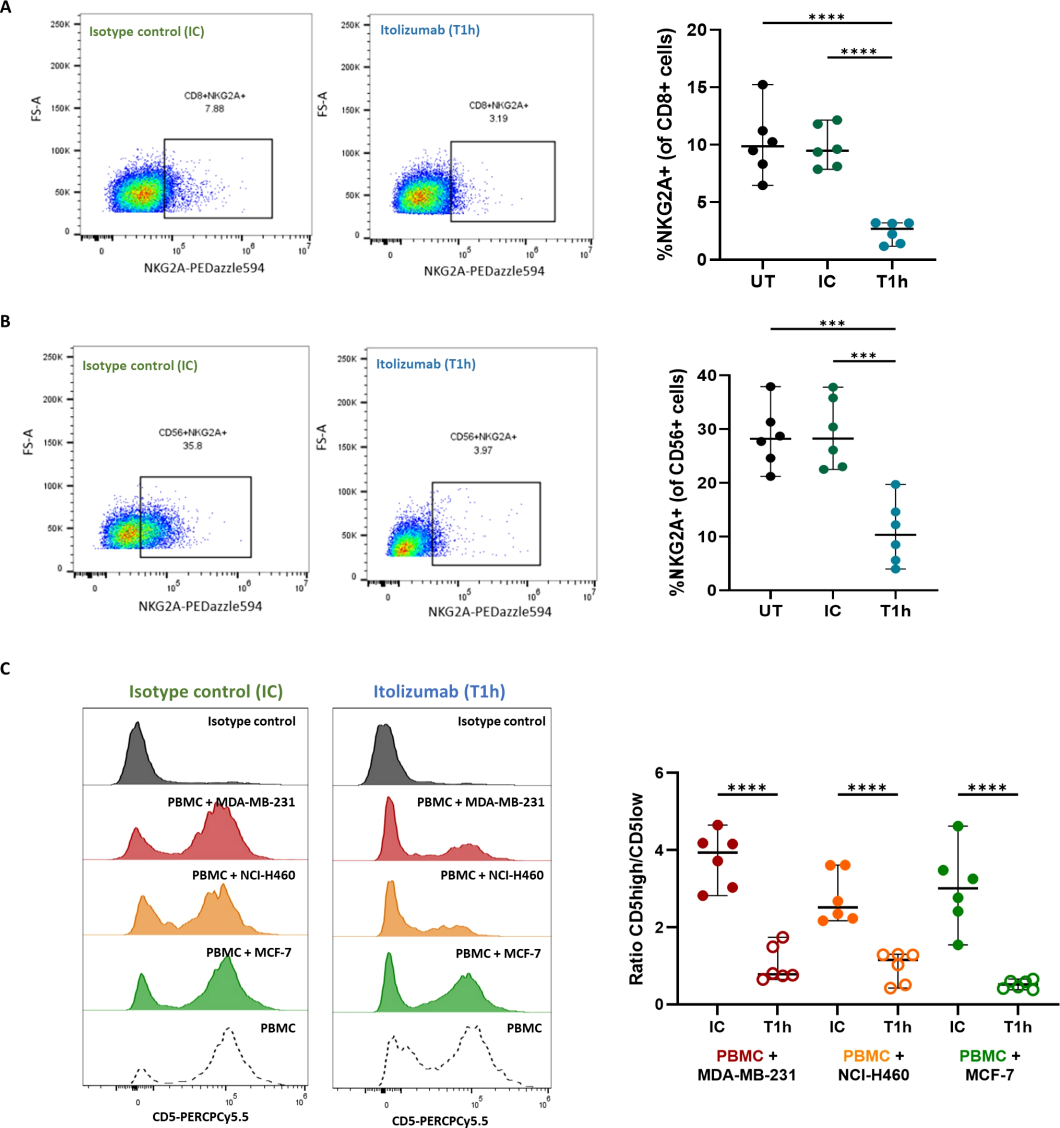
Itolizumab reduces the frequency of inhibitory receptors NKG2A within CD8+ T and NK cells and CD5 on T cells. Isolated CD8 T cells (n=6) or PBMC from healthy controls were maintained untreated (UT, black) or pre-incubated with 10 µg/mL isotype control (IC, green) or itolizumab (T1h, blue) and challenged with breast tumor cell line MDA-MB-231. Expression levels of NKG2A in **(A)** isolated CD8+ T and **(B)** NK cells were assessed by flow cytometry. Representative dot plots and the frequency of positive cells are shown. Statistical analysis was performed using one-way ANOVA with Tukey’s multiple comparisons test. **(C)** CD5 expression on isotype control and itolizumab-treated PBMC challenged with breast tumor cell lines MDA-MB-231 (n=6, red) and MCF-7 (n=6, green) and lung tumor cell line NCI-H460 (n=6, orange). Representative histograms of CD5 expression and ratio of CD5 high/CD5 low MFI on PBMC are displayed for each co-cultured. Data are depicted as median ± 95% confidence interval. Statistical analysis was performed using unpaired Student T-tests. Only statistical significance is shown in the graphs, with ***p ≤ 0.001, and ****p ≤ 0.0001.

Given that itolizumab induces activation on CD8+ T-cells and NK cells challenged with cancer cell lines, the effect of the antibody over inhibitory receptors in these subpopulations co-cultured with CD318+ tumor cell lines was investigated. Itolizumab significantly reduced the NKG2A expression in the CD8+ T-cells (**Figure 5A**) and NK cells (**Figure 5B**), inversely correlating with the NKG2D upraising.

Considering that CD5 is another inhibitory receptor involved in the negative modulation of the TCR signaling, as well as its high structural and functional homology with CD6 (28), CD5 expression on isotype control or itolizumab-treated T cells challenged with tumor cell lines was evaluated. NK cells do not usually express CD5, consequently, this cell type was excluded from the analysis. Unlike what was observed for CD6, confronting PBMCs with tumor cell lines does not modify CD5 expression on immune cells (**Figure 5C**), which suggests that despite its high structural homology with CD6, neither CD318 nor ALCAM had an impact on CD5 expression. Interestingly, in itolizumab-treated PBMCs in co-cultures, CD5 expression levels decreased without complete depletion (**Figure 5C**), which suggests a dual modulation of CD6 and CD5 levels in antitumoral T cell responses.

**Figure 6:**
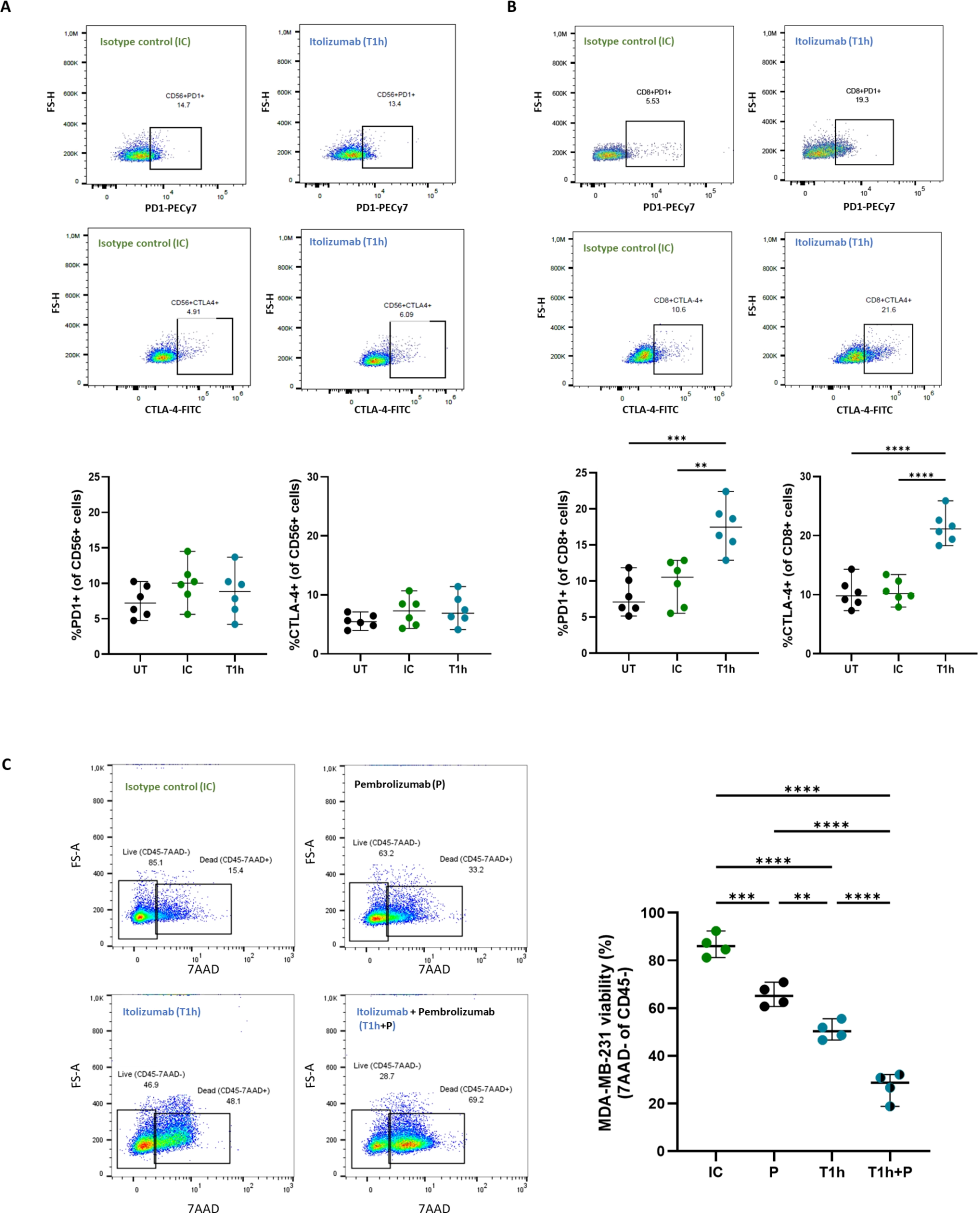
Increase of PD-1 and CTL-4 expression on CD8+ T cells by itolizumab promotes an additive cytotoxic effect in PBMC treated with combinations of itolizumab and other ICI. Isolated CD8+ T cells and NK cells (n=6) from healthy controls were maintained untreated (UT, black) or pre-incubated with 10 µg/mL isotype control (IC, green) or itolizumab (T1h, blue) and challenged with breast tumor cell line MDA-MB-231. Representative dot plots and frequency of PD1+ and CTLA4+ of **(A)** NK and **(B)** CD8+ T cells per donor are shown. **(C)** PBMC from healthy individuals were maintained untreated (UT, black dots) or pre-incubated with 10 µg/mL isotype control (IC, green dots), pembrolizumab (P, black dots), itolizumab (T1h, blue dots) or a combination of both (T1h+P, bicolor dots) and challenged with CD318+ breast tumor cell line MDA-MB-231. Tumor cell viability was measured by 7AAD staining by flow cytometry. Representative dot plots of 7AAD staining for each treatment and individual tumor cell viability percentages for each condition are displayed. Data are depicted as median ± 95% confidence interval. Statistical analysis was performed using one-way ANOVA and Tukey’s multiple comparisons test. Only statistical significance is shown in the graphs, with **p ≤ 0.01, ***p ≤ 0.001, and ****p ≤ 0.0001.

Interestingly, itolizumab maintains unchanged the expression frequency of PD-1 and CTLA-4 on NK cells (**Figure 6A**) and increases their expression on CD8+ T cells compared to isotype control (**Figure 6B**). CD8+ T and NK cells co-cultured with the other CD318+ cancer cell lines NCI-H460, HCT-116, and SKOV-3 displayed similar outcomes (**Supplementary Figure 4**).

### 3.6 Combinational treatment of itolizumab and pembrolizumab boosted cytotoxic effector cell functions

The increased cytotoxic capacity of PBMCs induced by blocking the CD6-CD318 association with itolizumab supports the notion that this interaction may represent an immune checkpoint similar to the PD-1/PD-L1 axis. Given the increased frequency of CD8+PD-1+ T lymphocytes observed in co-cultures in the presence of itolizumab and the clinical benefits reported for combinatorial therapies of different ICIs (29), the effect of the combination of itolizumab with pembrolizumab on PBMCs-induced tumor cell killing *in vitro* was evaluated. Determination of PD-L1 expression in MDA-MB-231 cell line and PD-1 expression in PBMC is shown in **Supplementary Figure 6**.

PBMCs challenged with the MDA-MB-231 line and treated with itolizumab showed higher cytotoxic capacity than those treated with isotype control and pembrolizumab (**Figure 6C**). However, the combination of both antibodies induced a significant additive increase in the cytotoxic capacity of PBMCs when confronted with the MDA-MB-231 tumor cells (**Figure 6C**), providing the first evidence for the successful use of itolizumab in combination with other ICI in the treatment of CD318+ solid tumors.

## 4 Discussion

Given the relevance of the interaction of CD6 and its ligands in the pathophysiology of autoimmune diseases and cancer, as well as the growing evidence of the role of modulation of these associations as revolutionary immunotherapies (3, 19, 20), this work aimed to evaluate whether CD6 modulation by the only clinically approved anti-CD6 antibody itolizumab improves the immune response against tumor cells. As a humanized antibody, itolizumab offers a clinically safer relevant therapeutic scenario for cancer treatment over other murine anti-CD6 antibodies.

*In vitro* treatment with itolizumab, enhanced tumor cell lines killing by PBMCs, similar to that reported for other murine anti-human CD6 antibodies (22) in different CD318+ tumor cell lines. Increased cancer killing was not induced by ADCC or complement-dependent cytotoxicity (CDC) as none of the tumor lines tested were CD6 positive, and itolizumab was proven not to induce ADCC or CDC (30). Allogeneic responses were also ruled out as increased cancer killing is only observed in the presence of itolizumab and not in untreated or isotype control-treated PBMC. In addition, CD6 negative cells have shown reduced reactivity to allogeneic stimulation (31, 32). Therefore, CD6 downmodulation by itolizumab in co-cultures reduces the likelihood of alloreactive responses.

The enhanced ability of itolizumab-treated effector cells to kill tumor cells implies that CD6 ligands on malignant cells negatively modulate cytotoxic cells through immunosuppressive mechanisms. ALCAM is the best-characterized and studied ligand of CD6 (15). Multiple studies have demonstrated the immunopathogenic role of CD6-ALCAM interaction in autoimmune diseases and cancer (33, 34). In the present work, improved tumor cell killing by itolizumab-treated PBMCs was not dependent on the blockade of CD6-ALCAM interaction.

Most antitumor therapies involving the CD6-ALCAM pathway exploit CD6’s role as a T-cell engager. The design of CAR-T cells with CD6 as a chimeric receptor has shown cytotoxic effects on ALCAM+ but not CD318+ human colon cancer cell lines (35, 36). However, blockade of the CD6-ALCAM interaction with anti-CD6 antibodies is an inhibitory therapy that reduces the effector capacity of immune cells (9). Patient selection with higher CD318+ and lower ALCAM+ tumors will be a critical step to improving the use of itolizumab as an immunotherapy against cancer.

Another CD6 ligand widely studied in cancer is CD318 (37). Overexpression of CD318 correlated with cancer cell growth and progression, metastasis formation, and worse prognosis in multiple types of malignancies (38, 39). The data explain the effect of itolizumab on increasing tumor cell line lysis by blocking CD6-CD318 interaction, as previously demonstrated (22). CD318 has been defined as a new molecular target for cancer immunotherapy, considering its role in the development and aggressiveness of tumors.

The present work shows an immunosuppressive effect of CD318 but not ALCAM on tumor cells, hampering proliferative responses of T cells and reducing immune cell viability and frequencies of CD6+ lymphocytes on co-cultures. CD6 downmodulation was observed in PBMCs co-cultured *in vitro* with tumor cell lines. However, since the effect was more pronounced in PBMCs challenged with CD318+ cell lines, it was postulated that the CD6-CD318 interaction rather than ALCAM-CD6 is responsible for the observable loss of CD6.

Several *in vivo* studies have demonstrated proteolytic cleavage of CD318 on tumor cells (40). Soluble forms of CD318 correlate with disease stage (41) and are associated with enhanced aggressiveness and metastatic potential of cancer cells (42, 43). However, in our *in vitro* experimental setup, CD318 cleavage does not appear to occur. Therefore, CD6 downmodulation in lymphocytes is not determined by the presence of soluble CUB1 domains.

In addition, the data provide the first evidence of a negative correlation between CD318 expression levels and CD6+ lymphocyte infiltration in breast cancer tissue samples. However, further research is needed to determine if the variability in CD6 and CD318 expression is associated with patient clinical characteristics and whether the inverse correlation between the molecules is driven by reduced tumor-infiltrating lymphocytes or downregulation of CD6 through interaction with CD318 in tumor cells.

Lower frequencies of CD6+ cells in PBMCs correlate with worse progression-free survival and overall survival in patients with non-small cell lung cancer and melanoma (44). Although a CD6 decline also occurs in the presence of itolizumab on co-cultures, the different response outcomes suggest that the mechanism involved in this lessening varies for the antibody regarding CD318. The differential modulation of CD6 levels on immune cells by CD318 and itolizumab could be explained by a cleavage mechanism that has recently been demonstrated for itolizumab (45). The antibody cleaves the extracellular domains of CD6, maintaining the cytoplasmic tail of the molecule (20), preserving CD6-dependent signaling and reducing apoptosis susceptibility (17). Furthermore, increased soluble CD6 (sCD6) generated by itolizumab could modulate antitumor lymphocyte effector function and tumorigenesis (21).

The blockade of CD318-negative signals does not exclude a direct activation mechanism of itolizumab on lymphocytes. Itolizumab-treated CD8+ T and NK cells expressed higher frequencies of activation markers and granzyme B and IFNγ production in monocultures as reported for UMCD6 (22, 23). More importantly, this effect was maintained when co-cultured with CD318+ tumor cells.

Augmented NKG2D expression in CD8+ T cells in the presence of itolizumab not only enhances antitumor responses against more aggressive MHC-deficient variants (43) but also generates CD6-ALCAM interaction-independent T cell activation, avoiding the inhibitory effects associated with blocking this interaction (44). Furthermore, higher levels of granzyme B and IFNγ are directly related to increased cytotoxic capacity, which correlates with better responses to ICIs and longer durations of clinical benefit (45–47).

However, while itolizumab activates cytotoxic lymphocytes challenged with MCF-7, it does not enhance cytotoxic capacity against these CD318 cell lines, reinforcing the theory that the increased tumor cell killing by itolizumab depends on the blockade of the CD6-CD318 interaction. The hypothesis that itolizumab restricts the immunosuppression of tumors on immune cells strengthens the evidence of the role of the CD6-CD6L interaction (mainly with CD318) as a potential target of ICI therapies.

Finally, it was evaluated whether the antitumor response rendered by itolizumab could also be associated with the modulation of inhibitory receptors, such as CD5, PD-1, or CTLA-4, on immune effector cells. CD6 and CD5 share a high degree of structural and functional homology. Both molecules are physically associated and are involved in TCR activating or inhibitory signal modulation (46). The downmodulation of CD6 and CD5 by itolizumab independent of CD318 expression suggests a regulatory mechanism of anti-tumor responses. CD5-targeted therapies have shown benefits in cancer treatment (47, 48), however, loss of CD5 is also associated with weaker antitumor responses (49). The fact that treatment with itolizumab does not entirely deplete CD5 in immune cells favors the maintenance of resistance to death induced by the activation of tumor-specific T lymphocytes, which offers an advantage over conventional therapies specific against CD5 (50).

The results also showed that itolizumab significantly enriched PD-1 and CTLA-4 expression on CD8+ T cells but not on NK cells challenged with CD318+ tumor cells. As itolizumab treatment broadens lymphocyte activation without completely depleting inhibitory receptors, using the antibody may mitigate the exacerbated immune responses often associated with ICI therapy (2). An increase in the frequency of PD-1+ and CTLA-4+ cells could also favor the design of combination therapies with other immune control inhibitors such as pembrolizumab or ipilimumab. In this regard, the present study demonstrates the additive potentiation of lymphocytes’ cytotoxic effects by combining itolizumab and pembrolizumab, which offers promising evidence for the potential benefits of combination strategies targeting CD6 and currently used checkpoint inhibitors.

The immunomodulatory effect of CD6 on immune cells, along with the dual role of the molecule in immune responses, is determined by the molecular interactions and signaling associated with its cytoplasmic tail and the interaction with its endogenous ligands (3, 19). The data demonstrates the effect of itolizumab in CD318+ cancer cell killing by lymphocytes while maintaining a level of inhibition in the cells that could prevent the exacerbation of the immune response commonly observed with ICI therapies.

## 5 Conclusions

Taken together, our results provide evidence linking the blocking of CD6 by the humanized anti-CD6 antibody itolizumab with the potentiation of functional properties of lymphocytes including cytotoxicity, proliferation, survival, and expression of checkpoint receptors in solid tumors, which highlight itolizumab as a novel clinically promising safer immunotherapy approach for CD318+ tumors and support the relevance of new combinatorial therapies with checkpoint inhibitors.

## Data Availability

The raw data supporting the conclusions of this article will be made available by the authors, without undue reservation.

## Supplementary material

Supplementary Figure 1 Optimization of tumor cell killing assays. (A) Kinetic tumor cell killing assays. MDA-MB-231, NCI-H460, SKOV-3, HCT-166, and MCF7 cell lines were seeded in a flat-bottomed 96-well culture plate (5x10^3^) and allowed to grow overnight. On the day of the assay, PBMCs were pre-incubated with 10 µg/ml of isotype control (IC, green lines) or itolizumab (T1h, blue lines) and added to tumor cells containing wells. The viability of tumor cell lines was measured by crystal violet staining after 24, 48, 72, 96, and 120 hours of co-culture. Greater statistically significant differences in the cytotoxicity of itolizumab-treated PBMC compared to the control isotype were observed after 120 hours of incubation. Images of crystal violet stained viable tumor cells in wells of co-cultures with IC (green frame) and T1h-treated PBMC (blue frame) are shown. (B) Dose-response curves. MDA-MB-231, NCI-H460, SKOV-3, HCT-166, and MCF7 cell lines were seeded in a U-bottomed 96-well culture plate (10^4^). PBMC were pre-incubated with 0, 0.1, 1, 10, and 100 µg/ml isotype control (IC, green lines) and itolizumab (T1h, blue lines) and added to wells containing tumor cells. The viability of the tumor cell lines was measured by flow cytometry after 120 hours of co-culture. Representative histograms are shown. After 120 hours of co-culture, greater statistically significant differences in cytotoxicity were observed in PBMC treated with 10 µg/mL itolizumab compared to other conditions. Data are depicted as mean ± standard derivation. Statistical analysis was performed using two-way ANOVA with Tukey’s multiple comparisons test (IC vs T1h for each co-culture time and antibody concentration). Only statistical significance is shown in the graphs, with *p ≤ 0.05, **p ≤ 0.01 and ***p ≤ 0.001.

Supplementary Figure 2 Human tumor cell lines and PBMC showed differential expression of CD6 and its ligands CD318 and ALCAM. (A) Histograms of expression levels of CD6, CD318, and ALCAM in tumor cell lines, and (B) individual values of MFI, and percentage of CD6 + cells in T lymphocytes (CD4+ and CD8+) and NK (CD56+) cells (n=10) were determined using flow cytometry. Data are depicted as mean ± 95% confidence interval. (C) Spearman correlation matrix of CD318 and ALCAM expression levels in all tumor cell lines with the tumor cell killing capacities of PBMC in co-cultures. The colors represent the strength of correlation, with dark green signifying a strong positive correlation and dark orange representing a strong negative correlation.

Supplementary Figure 3 Itolizumab downmodulates CD6 on immune cells. (A) Itolizumab-treated PBMC were cocultured with human tumor cell lines MDA-MB-231 (red), NCI-H460 (orange), and MCF-7 (green). CD6 loss was measured on PBMC using a non-competitive antibody by flow cytometry. Representative histograms of CD6 expression itolizumab-treated PBMC challenged with tumor cell lines are depicted. (B) CD6 loss in itolizumab-treated PBMC is not caused by receptor occupancy. After co-culture with MDA-MB-231 cells isotype control and itolizumab-treated PBMC were washed twice with flow cytometry solution (●) or glycine-HCL pH 2.5 solution (◼) to dissociate the CD6-T1h interaction. CD6 expression on PBMC was determined by flow cytometry using an anti-CD6 antibody (clone MEM98) with the same recognition epitope as itolizumab. Representative histograms of CD6 expression for each condition and frequency of CD6+ cells per donor are shown. Data are presented as median ± 95% confidence interval. Statistical analysis was performed using unpaired Student T-tests. Only statistical significance is shown in the graphs, with **p ≤ 0.01.

Supplementary Figure 4 Isolated CD8+ T and NK cells were pre-incubated with isotype control (IC, green dots) or itolizumab (T1h, blue dots) and challenged with lung (NCI-H460), ovarian (SKOV-3), and colon (HCT-116) cancer cell lines. NKG2D, PD-1,, and CTLA-4 expression on (A) CD8+ T lymphocytes and (B) NK cells were measured using flow cytometry. The percentage of positive cells per donor is shown. Data are depicted as median ± 95% confidence interval. Statistical analysis was performed using an unpaired Student T test between IC and T1h-treated PBMC. Only statistical significance is shown in the graphs, with *p ≤ 0.05, **p ≤ 0.01, ***p ≤ 0.001, and ****p ≤ 0.0001.

Supplementary Figure 5 Itolizumab-treated CD8+T and NK cells showed increased activation levels in monocultures and co-cultures with MCF-7 cell lines. Isolated CD8+T and NK cells were treated with 10µg/mL of isotype control (IC, green) or itolizumab (T1h, blue). Expression levels of NKG2D and CD69 were assessed on (A) monocultures and (B) in co-cultures with CD318- cell line MCF-7. Representative dot plots of each condition and individual percentage of positive cells are displayed. Data are depicted as median ± 95% confidence interval. Statistical analysis was performed using unpaired Student T-tests. Only statistical significance is shown in the graphs, with *p ≤ 0.05 and **p ≤ 0.01.

Supplementary Figure 6 (A) PD-L1 expression on MDA-MB-231 cell line. (B) Expression frequency of CD6 (n=20), CD5 (n=10), and PD-1 (n=10) on immune cells of healthy donors.
