# Supplementary figures and images for: Itolizumab regulates activating and inhibitory signals on effector cells, improving their cytotoxicity against CD318+ tumor cell lines
Source: Front Immunol. 2025 May 5;16:1585597. doi: 10.3389/fimmu.2025.1585597 (PMC12086168; doi:10.3389/fimmu.2025.1585597)

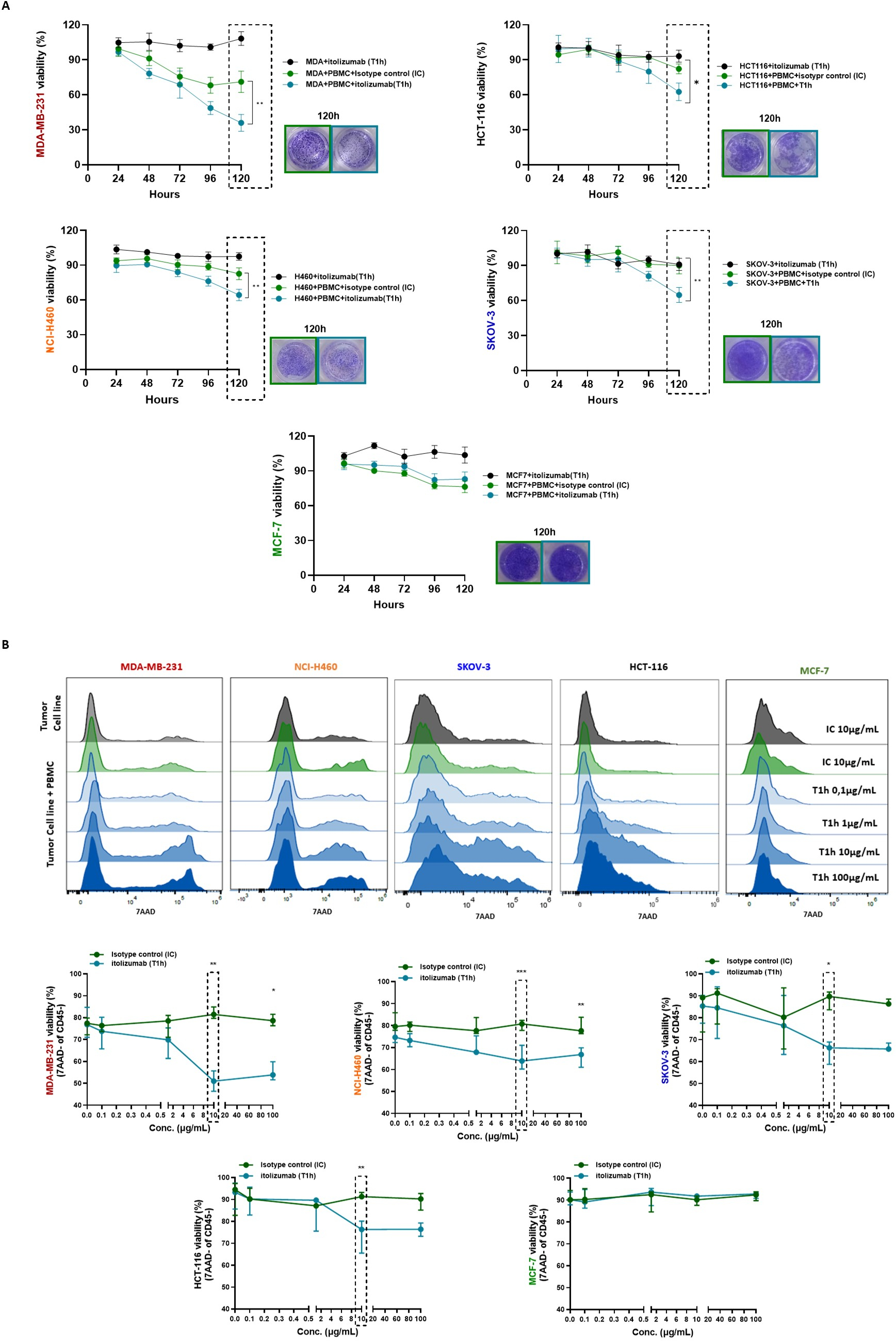

Supplement: Supplementary Figure 1 — Optimization of tumor cell killing assays. (A) Kinetic tumor cell killing assays. MDA-MB-231, NCI-H460, SKOV-3, HCT-166, and MCF7 cell lines were seeded in a flat-bottomed 96-well culture plate (5x10^3^) and allowed to grow overnight. On the day of the assay, PBMCs were pre-incubated with 10 µg/ml of isotype control (IC, green lines) or itolizumab (T1h, blue lines) and added to tumor cells containing wells. The viability of tumor cell lines was measured by crystal violet staining after 24, 48, 72, 96, and 120 hours of co-culture. Greater statistically significant differences in the cytotoxicity of itolizumab-treated PBMC compared to the control isotype were observed after 120 hours of incubation. Images of crystal violet stained viable tumor cells in wells of co-cultures with IC (green frame) and T1h-treated PBMC (blue frame) are shown. (B) Dose-response curves. MDA-MB-231, NCI-H460, SKOV-3, HCT-166, and MCF7 cell lines were seeded in a U-bottomed 96-well culture plate (104). PBMC were pre-incubated with 0, 0.1, 1, 10, and 100 µg/ml isotype control (IC, green lines) and itolizumab (T1h, blue lines) and added to wells containing tumor cells. The viability of the tumor cell lines was measured by flow cytometry after 120 hours of co-culture. Representative histograms are shown. After 120 hours of co-culture, greater statistically significant differences in cytotoxicity were observed in PBMC treated with 10 µg/mL itolizumab compared to other conditions. Data are depicted as mean ± standard derivation. Statistical analysis was performed using two-way ANOVA with Tukey’s multiple comparisons test (IC vs T1h for each co-culture time and antibody concentration). Only statistical significance is shown in the graphs, with *p ≤ 0.05, **p ≤ 0.01 and ***p ≤ 0.001. [file Image1.jpeg]

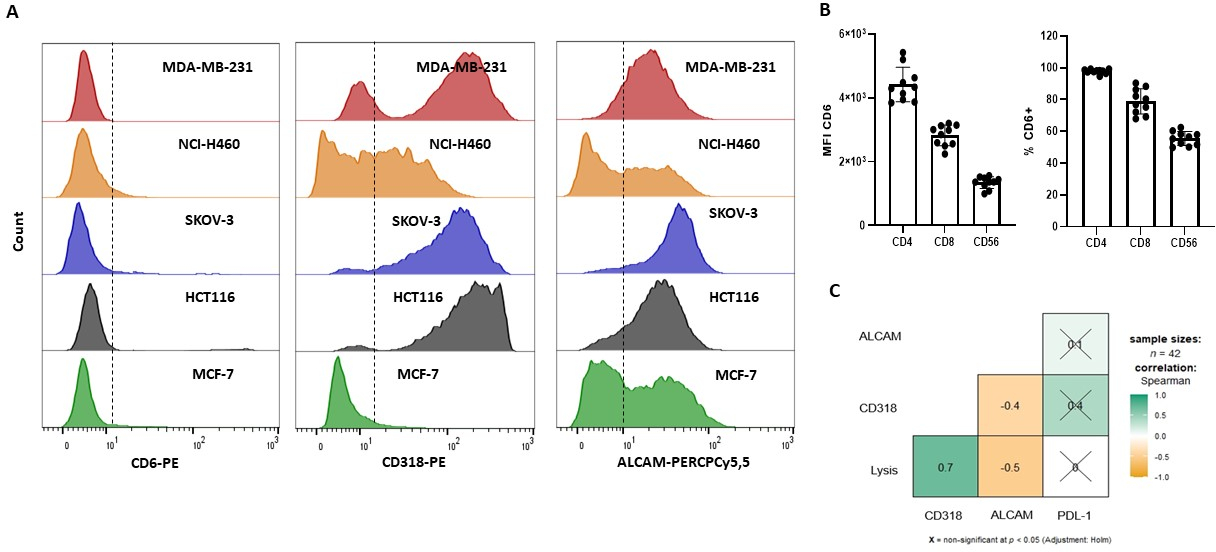

Supplement: Supplementary Figure 2 — Human tumor cell lines and PBMC showed differential expression of CD6 and its ligands CD318 and ALCAM. (A) Histograms of expression levels of CD6, CD318, and ALCAM in tumor cell lines, and (B) individual values of MFI, and percentage of CD6 + cells in T lymphocytes (CD4+ and CD8+) and NK (CD56+) cells (n=10) were determined using flow cytometry. Data are depicted as mean ± 95% confidence interval. (C) Spearman correlation matrix of CD318 and ALCAM expression levels in all tumor cell lines with the tumor cell killing capacities of PBMC in co-cultures. The colors represent the strength of correlation, with dark green signifying a strong positive correlation and dark orange representing a strong negative correlation. [file Image2.jpeg]

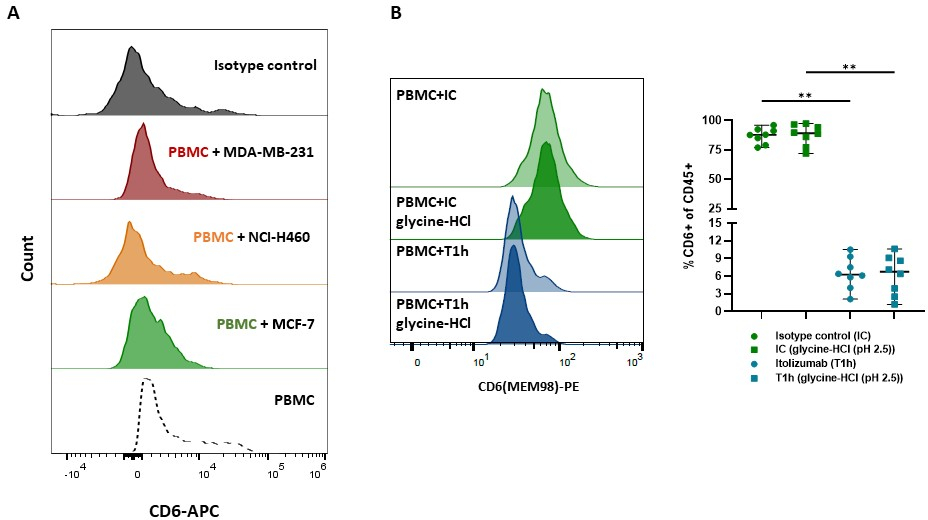

Supplement: Supplementary Figure 3 — Itolizumab downmodulates CD6 on immune cells. (A) Itolizumab-treated PBMC were cocultured with human tumor cell lines MDA-MB-231 (red), NCI-H460 (orange), and MCF-7 (green). CD6 loss was measured on PBMC using a non-competitive antibody by flow cytometry. Representative histograms of CD6 expression itolizumab-treated PBMC challenged with tumor cell lines are depicted. (B) CD6 loss in itolizumab-treated PBMC is not caused by receptor occupancy. After co-culture with MDA-MB-231 cells isotype control and itolizumab-treated PBMC were washed twice with flow cytometry solution (●) or glycine-HCL pH 2.5 solution (◼) to dissociate the CD6-T1h interaction. CD6 expression on PBMC was determined by flow cytometry using an anti-CD6 antibody (clone MEM98) with the same recognition epitope as itolizumab. Representative histograms of CD6 expression for each condition and frequency of CD6+ cells per donor are shown. Data are presented as median ± 95% confidence interval. Statistical analysis was performed using unpaired Student T-tests. Only statistical significance is shown in the graphs, with **p ≤ 0.01. [file Image3.jpeg]

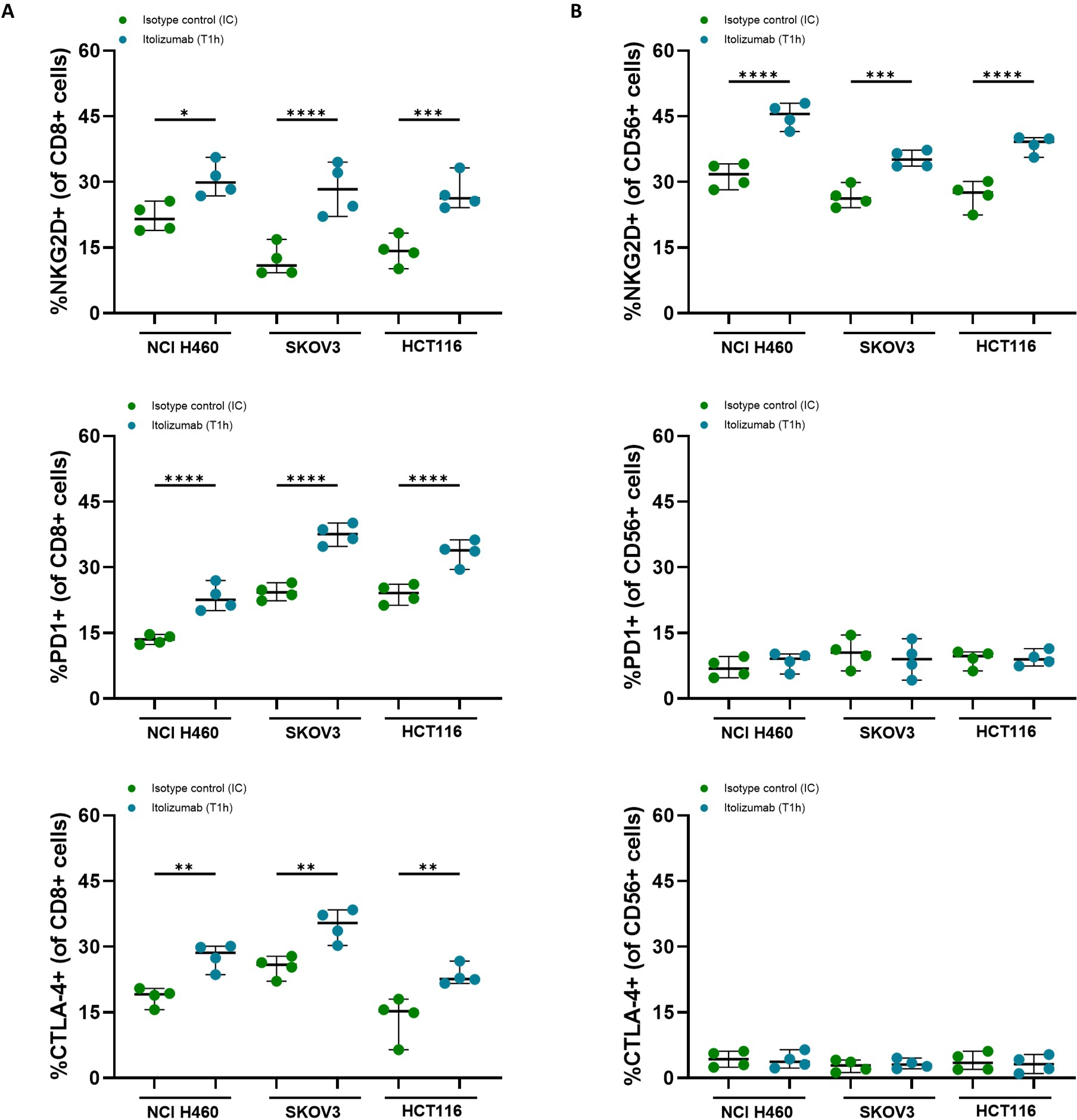

Supplement: Supplementary Figure 4 — Isolated CD8+ T and NK cells were pre-incubated with isotype control (IC, green dots) or itolizumab (T1h, blue dots) and challenged with lung (NCI-H460), ovarian (SKOV-3), and colon (HCT-116) cancer cell lines. NKG2D, PD-1,, and CTLA-4 expression on (A) CD8+ T lymphocytes and (B) NK cells were measured using flow cytometry. The percentage of positive cells per donor is shown. Data are depicted as median ± 95% confidence interval. Statistical analysis was performed using an unpaired Student T test between IC and T1h-treated PBMC. Only statistical significance is shown in the graphs, with *p ≤ 0.05, **p ≤ 0.01, ***p ≤ 0.001, and ****p ≤ 0.0001. [file Image4.jpeg]

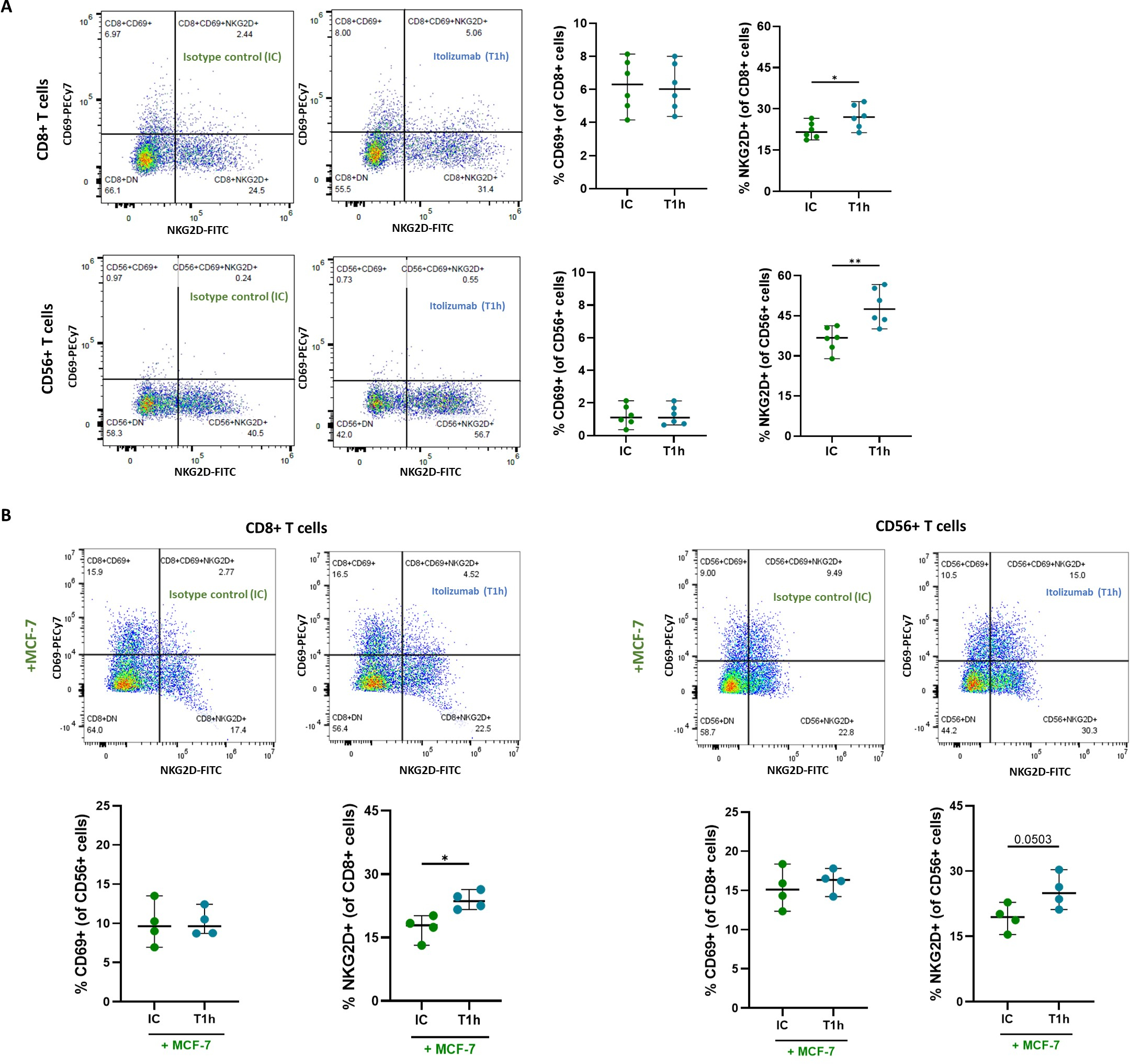

Supplement: Supplementary Figure 5 — Itolizumab-treated CD8+T and NK cells showed increased activation levels in monocultures and co-cultures with MCF-7 cell lines. Isolated CD8+T and NK cells were treated with 10µg/mL of isotype control (IC, green) or itolizumab (T1h, blue). Expression levels of NKG2D and CD69 were assessed on (A) monocultures and (B) in co-cultures with CD318- cell line MCF-7. Representative dot plots of each condition and individual percentage of positive cells are displayed. Data are depicted as median ± 95% confidence interval. Statistical analysis was performed using unpaired Student T-tests. Only statistical significance is shown in the graphs, with *p ≤ 0.05 and **p ≤ 0.01. [file Image5.jpeg]

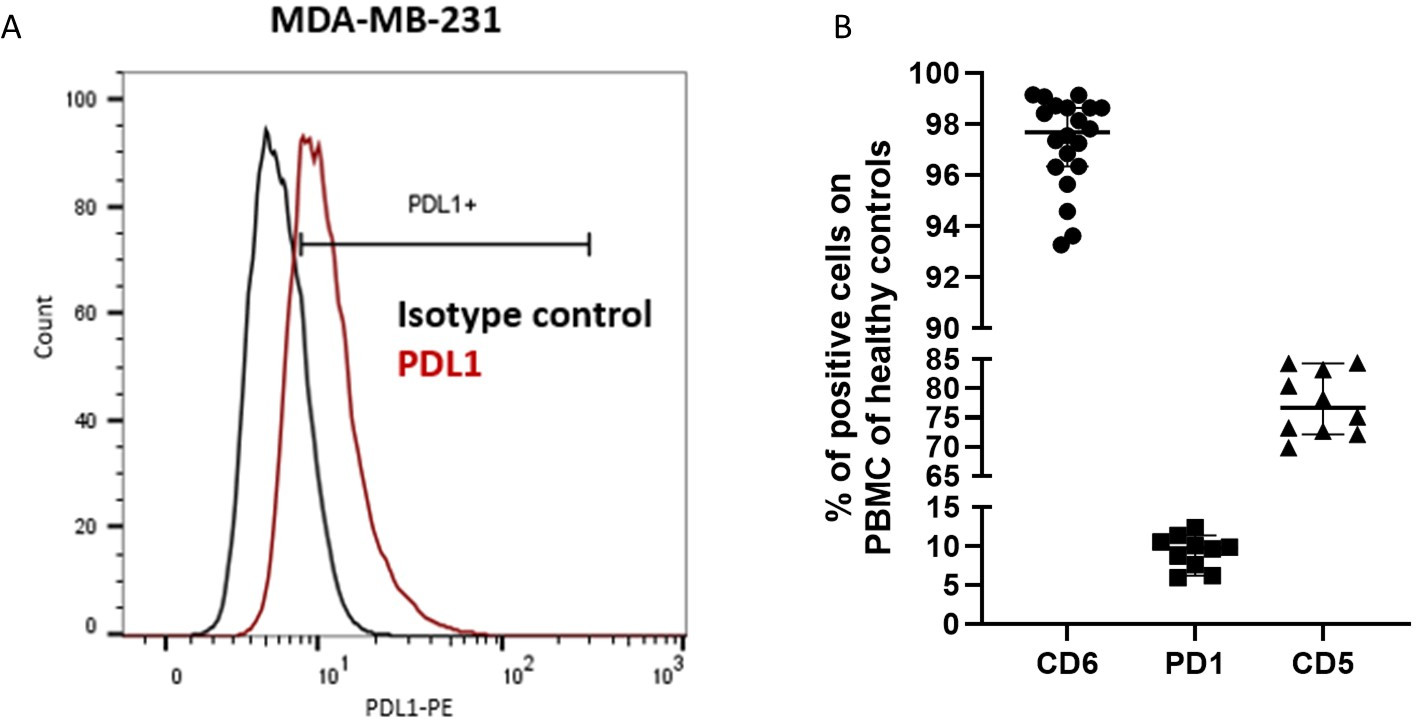

Supplement: Supplementary Figure 6 — (A) PD-L1 expression on MDA-MB-231 cell line. (B) Expression frequency of CD6 (n=20), CD5 (n=10), and PD-1 (n=10) on immune cells of healthy donors. [file Image6.jpeg]
