# Supplementary material for: Itolizumab regulates activating and inhibitory signals on effector cells, improving their cytotoxicity against CD318+ tumor cell lines
Source: Front Immunol. 2025 May 5;16:1585597. doi: 10.3389/fimmu.2025.1585597 (PMC12086168; doi:10.3389/fimmu.2025.1585597)
Supplement: Supplementary file 7 [file Table1.docx]

Supplementary Material

Supplementary Table 1. Clinical data of study subjects used for in vitro experiments and IHC evaluation.

|  | Healthy donors | Breast cancer patients  (IHC samples) |
| --- | --- | --- |
| Number | 32 | 117 |
| Age (years) | (25-48 years)  Mean 36.27 | (34-90 years)  Mean 58.35 |
| Female/male | 13/19 (0.68) | 117/0 (1) |
| Histological classification |  |  |
| Invasive ductal carcinoma |  | 64 |
| Invasive lobular carcinoma |  | 10 |
| Others |  | 13 |
| Clinical stage |  |  |
| IIa |  | 36 |
| IIb |  | 27 |
| IIIa |  | 13 |
| IIIb |  | 6 |
| IIIc |  | 1 |
